# Electrical activity of the masseter during swallowing after total laryngectomy

**DOI:** 10.1590/S1808-86942011000500018

**Published:** 2015-10-22

**Authors:** Leandro de Araújo Pernambuco, Hilton Justino da Silva, Gerlane Karla Bezerra Oliveira Nascimento, Elthon Gomes Fernandes da Silva, Patrícia Maria Mendes Balata, Veridiana da Silva Santos, Jair Carneiro Leão

**Affiliations:** 1 Master's degree in Health Science, Pernambuco Federal University (Universidade Federal de Pernambuco or UFPE). Assistant professor I of the Speech Therapy course, Rio Grande do Norte Federal University (Universidade Federal do Rio Grande do Norte or UFRN); 2 Doctoral degree in Nutrition, Pernambuco Federal University (UFPE). Adjunct professor II of the Speech Therapy course, Pernambuco Federal University (UFPE); 3 Master's degree student in Pathology, Pernambuco Federal University (UFPE). Speech therapist, State Health Office of Paraíba (Secretaria Estadual de Saúde da Paraíba); 4 Master's degree student in Human Health and the Environment, Pernambuco Federal University (UFPE). Clinical speech therapist; 5 Doctoral student in Neuropsychiatry and Behavioral Science, Pernambuco Federal University (UFPE). Speech therapist at the Human Resources Institute (Instituto de Recursos Humanos or IRH) of the Pernambuco State Government; 6 Master's degree in Biometrics, Pernambuco Rural Federal University (Universidade Federal Rural de Pernambuco or UFRPE). Assistant professor I at the Pernambuco Rural Federal University - Serra Talhada Academic Unit (UFRPE-UAST); 7 Doctoral degree in Oral Medicine, University of London (UL – England). Dental surgeon. Associate professor I at the Clinical and Preventive Dentistry Deparment (Departamento de Clínica e Odontologia Preventiva), Pernambuco Federal University (UFPE)

**Keywords:** electromyography, laryngeal neoplasms, masseter muscle, swallowing, swallowing disorders

## Abstract

**Abstract:**

Total laryngectomy is a surgical procedure that can change swallowing biomechanics, including muscle activity of the masseter; this muscle stabilizes the mandible.

**Aim:**

To characterize the electrical activity of the masseter muscle during swallowing after total laryngectomy. Series study.

**Material and Methods:**

An electromyographic evaluation of swallowing was carried out; three different volumes of water (14.5ml, 20ml and 100ml) were swallowed, and there was a rest condition. The electromyographic signal was normalized by Maximum Resisted Voluntary Activity - considered as 100% of electrical activity of muscles. All other values were calculated as a percentage of this parameter.

**Results:**

There is moderate electrical activity of the masseter during swallowing with higher averages on the left. There was no difference between swallowing 14.5ml or 20ml. Natural swallowing of 100ml had the lowest average. Electromyographic signals were recorded at rest on both sides, indicating the existence of electric activity in this situation.

**Conclusion:**

Patients submitted to total laryngectomy present electrical activity of the masseter muscles during swallowing and at rest. This activity is influenced by the volume of swallowed liquid, and showed significant differences among the tasks. *Clinical Trials*: NCT01095289

## INTRODUCTION

Given its important connection with the hyoid bone, the larynx has many movements and amplitudes and participates in several functions, such as swallowing^1^,^2^. The hyoid group elevates and moves forward when the food bolus passes from the mouth to the pharynx. At this moment, the masseter muscle works with the supra-hyoid muscles to fix and stabilize the mandible^3^,^4^.

Biomechanical changes may arise when laryngeal cancer is present; this disease comprises about 25% of head and neck tumors, it is more frequent in men in the fifth and sixth decades of life, and is strongly associated with smoking^5^,^6^.

The treatment of choice in extensive and infiltrating tumors is total laryngectomy, with or without radiotherapy and chemotherapy^7^. After surgery, the communication between the airway and digestive pathway becomes dissociated, and the trachea is implanted directly to the skin. Oropharyngeal dysphagia is one of the surgical complications^8^.

A study of the electrical activity of muscle groups involved in swallowing may help understand its biomechanics in post-total laryngectomy patients. Muscle electrical activity^5^ may be recorded with surface electromyography (sEMG), which is a method for recording variations in muscle electrical activity during contraction. This method evaluates the physiological and pathological status of muscles; in other words it yields data on the principles of muscle function, and may inform the diagnosis, and the prognosis^9^,^10^.

Descriptive data on the electrical activity of swallowing muscles in post-total laryngectomy patients are sparse in the literature; they may be an interesting basis for understanding the biodynamics of swallowing in these patients. The purpose of this study was to describe the electrical activity of the masseter muscle during swallowing in post-total laryngectomy patients.

## MATERIAL AND METHODS

The study population comprised 15 volunteers (14 male and 1 female; ages ranging from 45 to 70 years - mean = 56.93 years). All subjects were post-total laryngectomy patients with neck dissection and adjuvant radiotherapy at least 6 months and not more than 4 years before. Patients were selected from the outpatient clinic of the Speech Therapy Unit, Pernambuco Cancer Hospital.

During the sample enrollment period, subjects with pharyngocutaneous fistula, dehiscence, tissue necrosis or signs of infection, facial lymphedema that did not allow the masseter to be visualized and palpated, patients that were unable to understand simple commands, patients with previously confirmed neurologic, neuromuscular, or neurodegenerative disorders, individuals that had withstood head and neck trauma, patients with trismus, and subjects with signs and symptoms of temporomandibular joint dysfunction, were excluded from this study. The institutional review board of the Pernambuco Cancer Hospital approved this study (no. 43/2009). All volunteers signed a free informed consent form, as mandated by the Resolution MS/CNS/CNEP no. 196/96 of 10 October 1996.

A Miotool 200 (MIOTEC^®^, Sao Paulo, Brazil) fourchannel electromyography device coupled to a laptop was used for recording electromyographic signals. Signal processing was done using a data acquisition system with eight independent gains per channel (a 1,000 gain was used), a 20 Hz low-pass filter and a 500 Hz high-pass filter, two SDS500 sensors with connectors and reference cable - ground cable and a calibrator (Miotec^®^).

Surface child disposable electrodes were used (Meditrace^®^, Sao Paulo, Brazil); these are made of silverchloride silver (Ag-AgCl). A conductive gel was used to provide conductivity for the electromyography signal.

The skin was cleaned with gauze and 70^o^ alcohol to remove oil and other material that could increase impedance against signal pick-up. Skin hairs were removed with a shaver, as this procedure reduces impedance, increases the contact surface and improves signal quality - it was done only if subjects consented.

The electrodes were placed in a standard method starting with the reference (ground) electrodes, followed by the right side electrodes and then the left side electrodes. The reference electrode is used to minimize interference from external electrical noise; it was place far from the recording site of muscles, over the styloid process of the right arm. The other electrodes were placed in a bipolar configuration, over the masseter muscle belly, longitudinal to the muscle fibers. The masseter was located by asking subjects to perform occlusion at maximal resisted activity for three seconds; this made it possible to visualize and palpate the thickest portion of the muscle, the middle line of the muscle belly. The second electrode was placed 1.5 cm below the first, also longitudinal to the muscle fibers. The two unused channels were disabled.

Recordings were done in a room at the Speech Therapy Unit of a reference oncology hospital. The room was silent, at ambient temperature, and the lighting was artificial. Subjects remained seated comfortably on a chair with back support and no head support; the hands were placed over the thighs, the feet were flat on the floor, the head was erect and looking forward, according to the Frankfurt position. Subjects were unable to see the computer screen to avoid visual feedback, which would interfere with the assessment. Before testing, each volunteer was trained and given the necessary information.

Electromyography was done according to the following steps:

1 Maximum Resisted Voluntary Activity:^11^, ^12^, ^13^ subjects were asked to clench their teeth maximally for 5 seconds; this task was repeated three times with 10 second intervals between each contraction.
2 Rest (Rp): a single recording in the habitual position, with closed lips and no speech, chewing, or swallowing task for 60 seconds.
3 Swallowing a comfortable volume of liquid ^10^,^14^: swallowing water at ambient temperature in a single 14.5 ml swallow. Subjects were asked to place the water in the mouth, then hold for 3 seconds, and swallow upon the evaluator's command.
4 Swallowing an uncomfortable volume of liquid^10^,^14^: swallowing water, a single 20 ml swallow, which aimed to test the subject's adaptation to a larger water volume. Subjects were asked to place the water in the mouth, then hold for 3 seconds, and swallow upon the evaluator's command.
5 Continuous swallowing^10^,^14^: subjects were asked to continuously swallow 100 ml of water, as done habitually.

The software Miograph 2.0 (Miotec^®^, Sao Paulo, Brazil) was applied to present and interpret the signals; it transforms the raw signal into a root mean square, which shows the square root of the mean of squares of instantaneous recorded electomyographic traced signal amplitudes as a digitized signal expressed in microvolts (μV).

Electromyographic signals were analyzed based on a reference value (100%), which was the mean (in μV) of three repetitions in the maximum voluntary resisted activity, which is when subjects voluntarily recruit a significant number of muscle fibers. All other signals were defined as a percentage of this reference value in each subject. In the maximum voluntary resisted activity exercise, the first and the last seconds were eliminated, and only the intermediary 3 seconds were taken into account.

The mean of each one of three repetitions was calculated in the first two swallowing tasks; a final mean (in μV) was calculated for each channel. For natural swallowing, the mean muscle electric activity was calculated taking into account the entire swallowing time. At rest, the mean CVM during 60 seconds of recording time (in μV) was taken into account.

The means, recorded in μV, were transformed into percentage values relative to the reference value in each channel for each subject.

Descriptive statistical analysis (means and standard deviation) was done initially. Non-parametric statistics for paired data (Friedman's test) was applied to check for significant differences among the tasks (swallowing a comfortable volume of liquid, swallowing an uncomfortable volume of liquid, continuous swallowing, and rest); the significance level was 5%. Data analysis was done with the statistics software Stata v.10.0, Minitab v.15, and Estatbarto; the Microsoft Excel 2003 spreadsheet was used to build the tables.

## RESULTS

The data are shown in tables. Table 1 shows the mean and standard deviation of swallowing tasks bilaterally with different volumes and at rest. Data are expressed as percentages of the maximum voluntary resisted activity (100%) on each side.

**Table 1:** Distribution of electric activity of the masseter muscle during swallowing of different volumes and a rest in total laryngectomy patients.

	Tasks	N	Mean (%)	Standard deviation
	SWL 14.5 ml	15	25.47	18.30
	SWL 20 ml	15	25.89	17.53
RM	SWL 100ml	15	19.51	12.97
	Rest	15	6.53	3.30
	SWL 14.5 ml	15	30.12	17.82
	SWL 20 ml	15	30.50	16.06
LM	SWL 100ml	15	23.25	9.65
	Rest	15	9.22	7.36

RM: right masseter; LM: left masseter; SWL: swallowing.

Table 2 shows a comparison among tasks, based on the Friedman test.

**Table 2:** Comparison of the percentage means of electrical activity of the masseter muscle during swallowing and rest in swallowing tasks performed by post-total laryngectomy patients.

Tasks	SWL 14.5 ml	SWL 20 ml	SWL 100ml	Rest
Muscle				
RM	25.47AB	25.89B	19.51C	6.53D
LM	30.12EF	30.50FG	23.25G	9.22H

RM: right masseter; LM: left masseter; SWL: swallowing. The letters next to the means are the multiple comparisons in the Friedman test. The means or mean pairs with different letters indicate a significant difference (*p*<0.05) between the corresponding means.

Although the mean electrical activity of the masseter was higher after swallowing 20 ml, there was only a statistical difference when this mean was compared with the mean value at rest (bilaterally), and when this volume was compared with swallowing of 100 ml in the right masseter muscle. Swallowing 14.5 ml was statistically different from rest and continuous swallowing bilaterally; the mean electrical activity of the masseter was higher compared to these other two tasks. The mean electrical activity of the masseter was present at rest, which was lower compared to all swallowing tasks.

## DISCUSSION

It may be said that swallowing disorders following total laryngectomy are multifactorial, depending of the extension of surgery, the structures that were operated, the neopharyngeal reconstruction method, residual mobility of anatomical structures, tumor recurrence, decreased peristalsis, pharyngeal sensitivity, food residues in the neopharynx after swallowing, pseudodiverticulae due to a ruptured anastomosis, stenosis of the pharyngoesophageal segment, the effects of adjuvant or coadjuvant treatment (radiotherapy and chemotherapy), and comorbidities such as age. These factors may in some manner interfere with muscle activation patterns by altered the biomechanics of swallowing^8^,^15^, ^16^, ^17^, ^18^, ^19^.

Studies using sEMG and swallowing tests appear to investigate the submandibular region more often, in some cases neglecting the electric activity of the masseter during swallowing^20^, ^21^, ^22^. The electrical activity of the masseter muscle in swallowing has been studied in healthy populations, users of prostheses, and subjects with some forms of dysphagia^4^,^10^,^14^,^23^,^24^. However, no study on the electric activity of the masseter muscle in post-total laryngectomy patients has been published in the literature.

Such paucity of studies in the literature, as well as the variety of methods in sEMG studies, has made it difficult to discuss the results, and limits possible relations among studies.

Our data show that the masseter has electric activity during swallowing in the study population. The percentages are high relative to previous studies of normal subjects^24^, in which electric activity of the masseter muscle during swallowing of water (volume not informed) reached about 5% of the maximum voluntary contraction; this value is lower than those in our results and also lower than values encountered in previous studies made by our group with young healthy subjects^25^.

In absolute numbers, the mean values of the left masseter were higher than those of the right masseter muscle; this suggests an asymmetry, which may be physiological and compatible with normal function even in subjects not undergoing surgery^26^,^27^. Studies of sEMG have noted that there is masticatory preference in one side, which may generate different stimuli between sides and a swaying movement during chewing; this may lead to asymmetrical development of the facial skeleton and muscles^28^. It is not possible to state that our results are associated with total laryngectomy because of the small sample size and the influence of several factors which need to be better controlled in future studies.

It should be pointed out that the post-total laryngectomy volunteers did not undergo elevation and anteriorization of the hyolarynx, which suggests that activation of the masseter in these individuals may depend substantially of other mechanisms. An electromyiographic study found physiologic respiratory apnea during swallowing in subjects that supposedly would not present it because of total laryngectomy. The authors noted the influence of the swallowing nervous center on such apnea, suggesting that this physiologic mechanism may be preserved even after surgery because of plasticity of the system and its adaptation response^29^.

It is possible that the same occurs in swallowing, as the electromyographic signal may be affected by the anatomy and physiology of muscles, as well as peripheral nervous system control^30^.

It also seems that such activation may be strongly influenced by the type of laryngeal reconstruction. There is so far no clinical or scientific evidence about which pharyngeal reconstruction method yields the best swallowing response^19^, or about the precise adapted swallowing physiology following total laryngectomy^31^. In the present study, the type of reconstruction could not be controlled because of inconsistency in patient registries about this datum.

Another controversial point in the literature is the influence of dentition and its status on the electrical activity of swallowing muscles. Absence of teeth or the presence of dental appliances may add to changes in electromyographic potentials in the elevator and depressor muscles of the mandible; there is, however, no consensus among authors about the true impact of these changes on the mean electrical activity^32^, ^33^, ^34^, ^35^, ^36^, ^37^, ^38^, ^39^. Along these lines, we suggest controlling better the variable occlusal contact in future studies on swallowing, so that it may be correlated with other data.

It may be concluded that there was a significant difference between natural swallowing of water (100 ml) and the other tasks, on both sides. Swallowing of this volume yielded the lowest means among all swallowing tasks, as was the cases in a previous study of healthy subjects^10^.

This task differs from swallowing under command, because it is a more spontaneous task, and therefore closer to habitual swallowing. In this case there are different patterns of neurologic and peripheral muscle control between spontaneous swallowing and more voluntary activity of swallowing a specific volume under command^40^. It should be noted that the mean electrical activity was not affected by the swallowing volume, as there was no statistically significant difference between these tasks on both sides. Thus, it is possible to assume that the type of task execution may have a greater influence on the mean electrical activity of the masseter during swallowing that the volume.

Our results showed that electromyographic signals were present at rest, and that its mean relative to the maximum resisted voluntary activity was lower than the means in all swallowing tasks bilaterally. This finding is different from some published results in which the authors argue that electric muscle activity is absent during rest. According to these authors, the position of the mandible at rest is maintained by gravitational forces, tissue viscoelasticity, and negative intraoral pressure^24^,^41^.

On the other hand, other authors have found minimal electric activity in mandible elevator muscles, which appears to be controlled by sensory receptors and the central nervous system. Any change in the balance among these instances may change muscle electric activity^24^,^26^,^42^,^43^. This hypothesis seems appropriate to explain our results.

Other factors may have interfered with our results. The age group of the study population may have presented age-related changes^43^. There are also significant effects of chemoradiotherapy on swallowing^44^,^45^. Obviously these sequelae may affect muscle activation patterns considerably, and contribute to an altered or adapted swallowing pattern.

Our clinical experience and studies of human physiology suggest that post-total laryngectomy subjects maintain compensatory muscle patterns that configure morphofunctional and neuromuscular readaptations.

It is important to point out that compensation strategies may affect the electric activity of the masseter muscle, which may explain the significant differences among subjects when this muscle is assessed electromyographically during swallowing, underlining the fact that this function is a complex motor activity that recruits refined central control mechanisms^22^,^46^. This was a descriptive study, which limits its inferential reach. However, it has generated hypothesis that may be answered in future studies of different designs.

## CONCLUSION

We found that muscle electric activity is present in the masseter of post-total laryngectomy patients during swallowing; the highest means were found to the left. Such activity was affected by different forms of executing swallowing tasks. The highest means were found when swallowing a specific volume under command. Continuous swallowing, a more spontaneous and physiologic task, had the lowest muscle electric activity means among all swallowing tasks. Electromyographic signals were recorded at rest on both sides, indicating the existence of electric activity in this situation.
